# The Effect of Self-Compatibility Factors on Interspecific Compatibility in *Solanum* Section *Petota*

**DOI:** 10.3390/plants12081709

**Published:** 2023-04-20

**Authors:** William L. Behling, David S. Douches

**Affiliations:** Department of Plant Soil and Microbial Sciences, Michigan State University, East Lansing, MI 48824, USA; behling3@msu.edu

**Keywords:** self-compatibility, interspecific, *S-RNase*, *Sli*

## Abstract

The relationships of interspecific compatibility and incompatibility in *Solanum* section *Petota* are complex. Inquiry into these relationships in tomato and its wild relatives has elucidated the pleiotropic and redundant function of *S-RNase* and *HT* which tandemly and independently mediate both interspecific and intraspecific pollen rejection. Our findings presented here are consistent with previous work conducted in *Solanum* section *Lycopersicon* showing that *S-RNase* plays a central role in interspecific pollen rejection. Statistical analyses also demonstrated that *HT-B* alone is not a significant factor in these pollinations; demonstrating the overlap in gene function between *HT-A* and *HT-B*, as *HT-A*, was present and functional in all genotypes used. We were not able to replicate the general absence of prezygotic stylar barriers observable in *S. verrucosum*, which has been attributed to the lack of *S-RNase,* indicating that other non-*S-RNase* factors play a significant role. We also demonstrated that *Sli* played no significant role in these interspecific pollinations, directly conflicting with previous research. It is possible that *S. chacoense* as a pollen donor is better able to bypass stylar barriers in 1EBN species such as *S. pinnatisectum*. Consequently, *S. chacoense* may be a valuable resource in accessing these 1EBN species regardless of *Sli* status.

## 1. Introduction

The value of wild species in the genetic improvement of potato was first demonstrated over a century ago in response to late blight epidemics in the northern hemisphere [1]. Unfortunately, several barriers inhibit efficient introgression of traits from wild species. These barriers can be largely separated into three categories: differences in ploidy, prezygotic barriers, and post-zygotic barriers [2]. Here, we will examine prezygotic interspecific barriers associated with gametophytic self-incompatibility in potato. 

The 107 wild species of potato represent a rich and diverse source of disease resistance and tuber quality traits for cultivated potato [2,3]. However, in the century-long history of potato breeding the enormous value of wild species traits has been realized only occasionally [1]. Among all the species characterized, the diploid 1 Endosperm Balance Number (1EBN) species are of particular interest, as they exhibit exceptional resistance to economically important pests such as early blight (*Alternaria solani*), Colorado potato beetle (*Leptinotarsa decemlineata*), and late blight (*Phytophthera infestans*) [4,5,6]. However, due to prezygotic stylar barriers and differences in effective ploidy between 1EBN species and cultivated germplasm, accessing the traits from these species is extraordinarily difficult [2,7,8]. Here, we seek to clarify the role of prezygotic barriers that prevent the use of 1EBN species in diploid potato breeding schemes.

Gametophytic self-incompatibility (GSI) is the default condition in diploid cultivated potato and most of its wild relatives [9,10,11]. GSI in Solanaceae is governed by a the multiallelic *S*-locus on chromosome 1, containing *S-RNase* tightly linked to multiple *SLF* genes [12,13,14]. This GSI system also serves the dual function of preventing interspecific pollinations [15,16]. This is accomplished by inhibiting the growth of self and interspecific pollen tubes, which serve as vehicles delivering male gametes to the female gametophyte, thus preventing fertilization. Because of this general overlap in function, interspecific crosses in Solanaceae often display unilateral incompatibility or incongruity (UI). UI usually follows the self-incompatible (SI) × self-compatible (SC) rule reported by Lewis and Crowe [17] where SC species can only act as the female, as the functional GSI system in the SI species prevents self and interspecific pollen from reaching the ovary [15,16,18]. While the SI × SC rule proposed by Lewis and Crowe [17] is generally helpful, it is an oversimplification and does not fully capture the complex relationships of interspecific compatibility and incompatibility in *Solanum*. Interspecific pollen rejection by SC species has been observed between members of the tomato and potato clades of *Solanum* [15,19,20]. This can be partially explained by the genetic differences in gene presence and function between SC species. GSI is primarily mediated by *S-RNase* and other components such as the asparagine-rich HT proteins which facilitate the function of S-RNase [15,20,21,22,23]. The lack of functional *S-RNase* alleles will result in SC and greater acceptance of interspecific pollen [12,20]. Additionally, *HT* has been demonstrated to mediate SC as well as *S-RNase*-dependent and independent interspecific compatibility [20,23,24].

### SC Factors Tested

**Figure 1:**
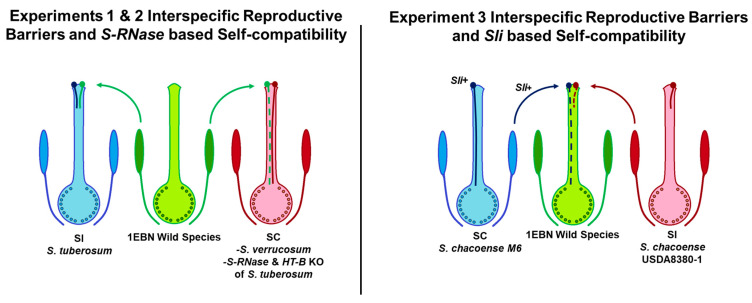
Experimental approach: diagram of the crosses made in these experiments. Solid lines representing pollen tube growth indicate known behavior, while dashed lines represent hypothesized behavior. In experiments 1 and 2 The 1EBN wild species *S. bulbocastanum*, *S. commersonii*, *S. jamesii*, and *S. pinnatisectum* were used as pollen donors. In experiment 1, wild-type DRH195 (*S. tuberosum*) was used as the self-incompatible (SI) control and compared with CRISPR-Cas9 knock-outs of *S-RNase* and *HT-B* in DRH195. Experiment 2 evaluated interspecific reproductive barriers in *S. verrucosum*, where the self-compatibility (SC) factors are not fully understood. In experiment 3, the *S. chacoense* clones M6 and USDA8380-1 were used as pollen donors since the SC factor being tested (*Sli*) is pollen expressed.

Two different mechanisms are used as sources of SC in potato, *S-RNase* based, and *S*-locus inhibitor (*Sli*) based SC. *S-RNase* based SC aims to remove barriers by the introduction of dysfunctional *S-RNase* alleles from wild species or knocking out functional alleles via gene editing [12,25]. *S-RNase* based SC can also be supplemented by the addition of dysfunctional alleles of *HT* and possibly other factors [20,23,26]. Central to the GSI system, the function of *S-RNase* is to degrade the RNA of incompatible pollen tubes. In plants with non-functional *S-RNase* such as tomato, the central barrier to self-pollen is eliminated, resulting in SC and broad interspecific compatibility (Figure 1) [15,20,23]. While *S-RNase* based SC aims to eliminate barriers associated with GSI, *Sli*-based SC hijacks the GSI system to achieve SC. In functional GSI systems a suite of 16–20 SLF proteins act as part of a collaborative non-self-recognition system to degrade non-like S-RNase proteins [13,27]. Sli is a non-S-locus F-box protein located on chromosome 12 capable of interacting with a wide range of *S-RNase* variants [27]. Thus, the presence of *Sli* in self-pollen initiates the degradation of S-RNase and confers SC even when the GSI system is still functional (Figure 1) [27,28].

The ability of *Sli* to overcome interspecific reproductive barriers (IRBs) was first reported by Sanetomo et al. [29], and the role of *HT* and *S-RNase* in IRBs has been demonstrated in Solanum section Lycopersicon by Tovar-Mendez et al. [23]. In potato, *S. verrucosum* is well recognized for its SC and lack of general IRBs [2,19,30]. The strong SC and lack of IRBs observable in *S. verrucosum* is likely rooted in its lack of a functional S-RNase protein [25,31]. While it is known that there is no detectable S-RNase on the protein level, it is unknown if the alleles for *S-RNase* in *S. verrucosum* are missing, non-functional, or inhibited in some way, or if *HT* or other factors play a role in these interactions [31].

The two objectives in this study were to: First, characterize the role of *S-RNase* and *HT* in the IRBs between cultivated *S. tuberosum* as the female and the wild species *S. bulbocastanum*, *S. commersonii*, *S. jamesii*, and *S. pinnatisectum* as pollen donors. CRISPR-Cas9 gene knockouts of *S-RNase* and *HT-B* in the *S. tuberosum* clone DRH195 were used to examine each of these factors independently and together. These results were compared with *S. verrucosum* as the female to see if knocking out *S-RNase* and *HT-B* replicated the phenotype observed in *S. verrucosum*. Second, in order to better capture the value of *Sli*, a set of crosses was designed to confirm the findings of Sanetomo et al. [29] that *Sli* contributes to interspecific compatibility and determine if these findings can be applied to other species. The ability of *Sli* to overcome IRBs could be an important factor in accessing the sexually isolated 1EBN species [29]. Additionally, the widespread use of *Sli* based SC in North American diploid potato breeding would mean that most public and private breeders would have access to these methods [32].

## 2. Results

### 2.1. The Role of S-RNase and HT-B in Interspecific Pollen Rejection

In order to determine the contributions of *S-RNase* and *HT-B* in interspecific pollinations, we utilized independent and dual CRISPR-Cas9 knockouts (KO) of *S-RNase* and *HT-B* of the *S. tuberosum* clone DRH195 as females in this experiment (Table 1). The wild species *S. bulbocastanum*, *S. commersonii*, *S. jamesii*, and *S. pinnatisectum* were used as pollen donors to test the strength of the IRBs in the female lines used. Pollinations were made using fresh pollen on newly opened flowers, and styles were collected for pollen tube measurements 48 h post pollination.

**Figure 2:**
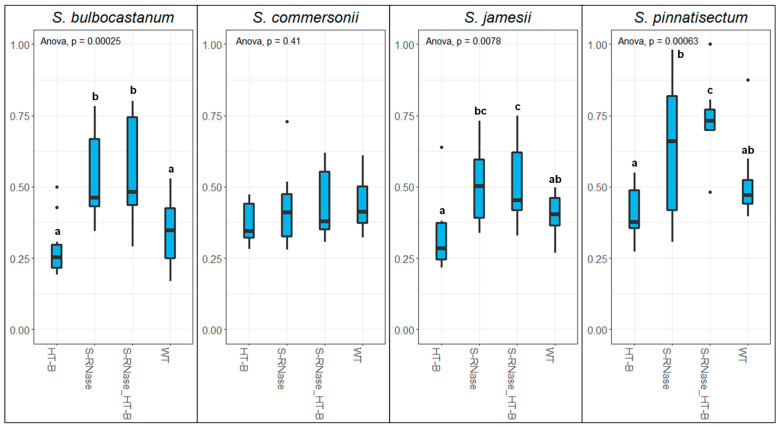
Measurement of the longest pollen tube as a proportion of total style length at 48 h post-pollination for all pollen donors. Female knockouts and wild type DRH195 (*S. tuberosum*) genotypes are depicted on the *x* axis, pollen donor species are listed at the top of each graph, and the style length is depicted on the *y* axis. Letters indicate statistically significant differences.

Stylar squash assays were employed to visualize the growth and inhibition of pollen tubes within the style. Results from statistical analysis of pollen tube growth are consistent with previous findings in tomato showing that *S-RNase* plays a central role in interspecific pollen rejection [23]. This is readily observable in the growth of pollen tubes from *S. bulbocastanum* and *S. pinnatisectum*. Pollen tubes from these two species were able to penetrate significantly further down the styles in the *S-RNase* KO of DRH195 compared to the wild-type. Additionally, when *S. bulbocastanum* and *S. pinnatisectum* were the pollen donors, the longest pollen tubes were nearly able to traverse the entire length of the style in *S-RNase* knockouts. With *S. jamesii* and *S. commersonii* as the pollen donors the effect of *S-RNase* was not statistically significant, although the inhibitive effect on pollen tube growth of *S-RNase* was still evident for *S. jamesii* (Figure 2). It should be pointed out that in pollinations with *S. pinnatisectum*, the difference between pollen tube growth in the *S-RNase* KO vs the *S-RNase*/*HT-B* KO was statistically significant, but not practically significant. Pollen tube penetrance in these pollinations was highly variable, and although a few pollen tubes from *S. pinnatisectum* were able to reach the ovary, no fruit development was observed.

Results from the same analyses demonstrate that *HT-B* alone is not a significant factor in the inhibition of pollen tubes from these species. No significant difference was observed between the ability of pollen tubes to penetrate the styles of wild-type *S. tuberosum* compared to the *HT-B* KO. Likewise, the differences between the *S-RNase* KO and the *S-RNase*/*HT-B* KO were not significant; showing that knocking out both *S-RNase* and *HT-B* compared to *S-RNase* alone offered no advantage to pollen tubes attempting to traverse the style. For example, pollen tubes from *S. jamesii* were generally arrested closer to the stigma in the *HT-B* KO compared to the wild type *S. tuberosum* (Figure 2). These results do not contradict the findings from Tovar-Méndez et al. [20,23] where HT proteins did play a significant role in interspecific pollen rejection. Due to the vicissitudes of gene editing, KOs of *HT-A* were not available during this study. Since *HT-A* remains functional and active, with its expression in DRH195 proven by RT-PCR and cDNA sequencing in all of the female genotypes used, it can be concluded that either: (1) *HT-A* plays a greater role in interspecific pollen rejection compared to *HT-B*; (2) there is a significant overlap in gene function between *HT-A* and *HT-B* so the loss of *HT-B* is not significant; or (3) *HT* in general is not a significant component in interspecific pollen rejection in these combinations.

The contrast between *S. commersonii* and the other pollen donors highlights the diversity in interspecific pollen rejection systems. *S. commersonii* was unique as a pollen donor as both *S-RNase* and *HT-B* did not appear to affect the rejection of pollen in the styles of DRH195. As discussed previously, since *HT-A* is still functional, *HT-A* alone may mediate the *S-RNase/HT-B* independent rejection of *S. commersonii* pollen in these pollinations. If this is the case, this would be consistent with the findings of Tovar-Méndez et al. [20]. The rejection or allowance of interspecific pollen is entirely dependent on the specific combination of species used as parents [15]. This is also the case with the role of certain genes in interspecific pollen rejection. *HT-A*, *HT-B*, *S-RNase*, and other factors such as the 120 kDa arabinogalactan protein or farnesyl pyrophosphate synthase (*FPS2*) may play significant or inconsequential roles during the rejection of interspecific pollen, and these roles are entirely dependent on the specific species and genotypes present in these interactions [15,16,33].

### 2.2. Prezygotic Interspecific Barriers in S. verrucosum

*S. verrucosum* was used as a female in this experiment, with *S. bulbocastanum*, *S. commersonii*, *S. jamesii*, and *S. pinnatisectum* as pollen donors. The same pollination protocols were used for all experiments and are detailed in the materials and methods. *S. verrucosum* is well recognized for its SC and lack of IRBs which can partially be explained by the lack of a functional S-RNase protein [2,19,25,30,31]. Because of the lack of IRBs, the results from this experiment were considered as the benchmark for compatible interspecific pollinations with these pollen donors.

**Figure 3:**
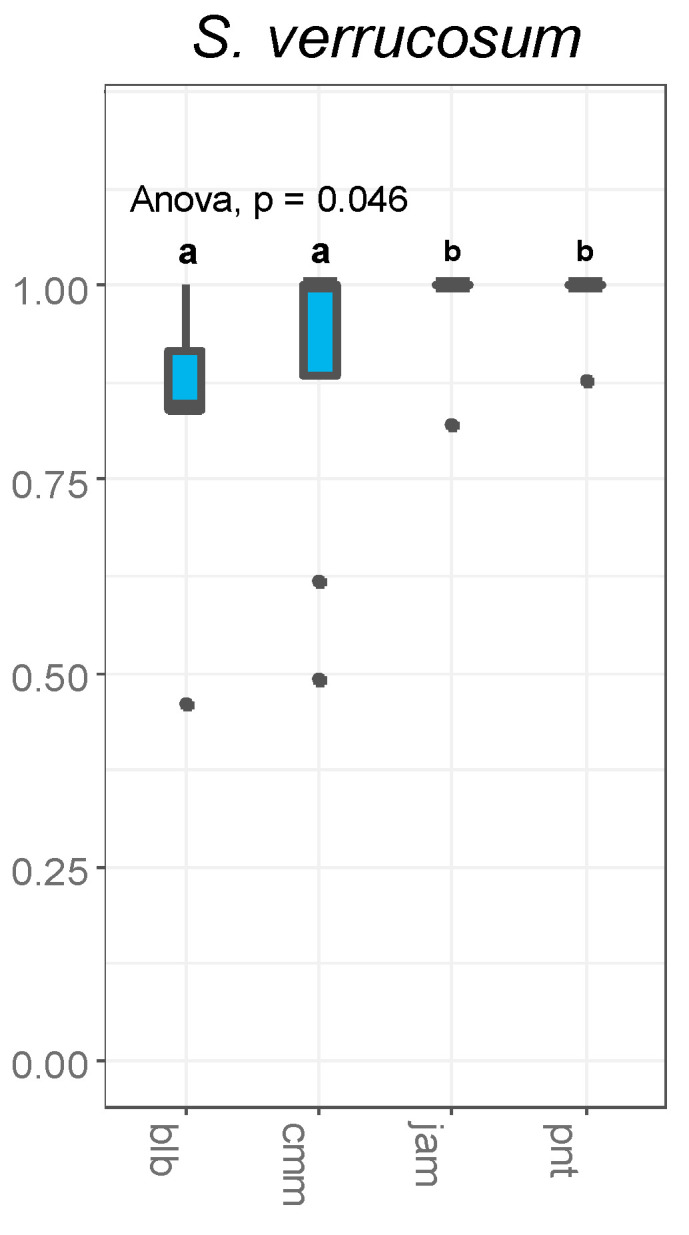
Measurements of the longest pollen tube for all pollen donors as a proportion of total style length at 48 h post-pollination. *S. verrucosum* was used as the female in all pollinations. Pollen parents are depicted on the *x* axis, and the style length is depicted on the *y* axis. Letters indicate statistically significant differences. Pollen donor species are represented by the following three letter species codes: *S. bulbocastanum* (blb), *S. commersonii* (cmm), *S. jamesii* (jam), and *S. pinnatisectum* (pnt).

In *S. verrucosum*, pollen tubes from all pollen donors, except for *S. bulbocastanum*, were able to easily travel down the full length of the style (Figure 3). Pollen tubes from *S. jamesii* and *S. pinnatisectum* were always present in the ovary at 48 h post pollination. The majority of pollen tubes from *S. bulbocastanum* did not penetrate the full length of the style. Despite this difference, fruit formation was observed for all four pollen donor species and hybrid progeny between *S. verrucosum* and the pollen donors *S. bulbocastanum* and *S. commersonii* were recovered. Overall, no significant stylar barriers were observed and pollen tubes from all four pollen donors were observed in the vicinity of the ovules.

### 2.3. The Role of Sli in Interspecific Pollen Rejection

*Sli* operates differently than the SC mechanisms in the previous experiments. Knocking out pistil expressed *S-RNase* and *HT-B* renders the GSI system non-functional, effectively removing the barrier to self-pollen [12,20,23,26]. *Sli* is expressed in the pollen and initiates the degradation of S-RNase to confer SC in functional GSI systems [27,28]. Because of this, the direction of the crosses in this experiment are reversed from the previous two. The wild species *S. bulbocastanum*, *S. commersonii*, *S. jamesii*, and *S. pinnatisectum* were used as females, and the two *S. chacoense* clones M6 and USDA8380-1 (80-1) were used as pollen donors. M6 (*Sli* +/+) is a homozygous, well characterized, and widely used source of *Sli*-based SC [32,34]. In order to reduce confounding genetic variables, clone 80-1 (*Sli* −/−) was selected as the non-*Sli* control as it is largely homozygous, and also the same species as M6 (*S. chacoense*) [35].

**Figure 4:**
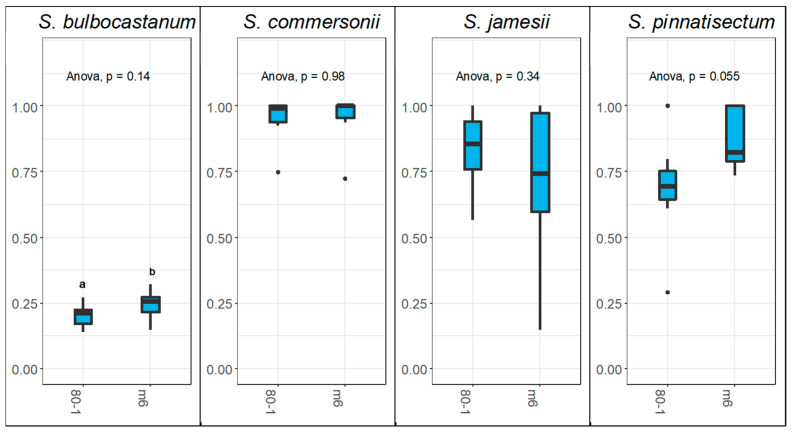
Measurement of the longest pollen tube according to female as a proportion of total style length at 48 h post pollination. Female genotypes are titled at the top of each graph. Pollen parents M6 (*Sli* +/+) and 80-1 (*Sli* −/−) are depicted on the *x* axis, and the style length is depicted on the *y* axis. Letters indicate statistically significant differences.

A statistically significant difference was observed between *Sli* +/+ and *Sli* −/− pollen donors in the styles of *S. bulbocastanum* with pollen tubes of the *Sli* +/+ clone M6 traveling further down the style (Figure 4). However, the same analysis failed to find any statistical significance between pollen donors in the styles of *S. commersonii*, *S. jamesii*, or *S. pinnatisectum*, which is inconsistent with the findings in previous research [29]. Pollen tubes from M6 generally traveled further down the style in *S. bulbocastanum*, *S. commersonii*, and *S. pinnatisectum* compared to pollen tubes from the SI (*Sli* −/−) clone 80-1 (Figure 4). However, even the statistically significant differences between pollen donors failed to have any practical significance, and did not change the outcome of these interspecific pollinations. For example, in the styles of *S. bulbocastanum* pollen tube growth from 80-1 was arrested closer to the stigma, while the majority of pollen tubes from M6 penetrated much further down the style. But, since all pollen tubes from both pollen donors were arrested in the first third of the *S. bulbocastanum* styles, this difference lacked any practical significance. *Sli* presence or absence did not have a significant impact on interspecific pollen-tube inhibition in the styles of *S. commersonii*, *S. jamesii*, and *S. pinnatisectum*. Pollen tubes from M6 generally grew further down the style in *S. pinnatisectum* with the opposite being true for *S. jamesii*, but these differences between pollen donors lacked significance. Unlike the other species combinations analyzed, there was a substantial amount of variability in the growth of M6 pollen tubes in *S. jamesii* styles. It isn’t clear why this is the case, but it may be attributable to unknown environmental factors. No pollen tubes from either pollen donor entered the ovary in *S. jamesii*, while most pollen tubes from both pollen donors traversed the entire style in *S. commersonii* and *S. pinnatisectum*. Pollinations of *S. pinnatisectum* by both M6 and 80-1 also initiated fruit set, although all the seeds were aborted likely due to differences in effective ploidy.

The presence or absence of *Sli* did not change the outcome of any of the pollinations made. Additionally, no significant difference was observed in pollinations made with *S. pinnatisectum*, which is inconsistent with the findings of Sanetomo et al. [29] despite the use of the same *S. pinnatisectum* PI (275232). The lack of consistent findings between these experiments and those of Sanetomo et al. [29] is likely attributable to some unknown factors in pollen donors besides *Sli*. The pollinations conducted in this study demonstrated that the clones M6 and 80-1 were comparable in their ability to bypass stylar barriers, or in their uniform inhibition. Unlike the pollen donors used by Sanetomo et al. [29] these pollen donors were of one species, *S. chacoense*, while the pollen donors used by Sanetomo et al. [29] came from interspecific crosses between *S. chacoense* and *S. phureja*. The difference between pollen donors is the likely source of inconsistencies; the introduction of unknown genetic variables from *S. phureja* may have introduced confounding genetic variables. The ability to form viable seeds cannot be assumed to be attributable to the presence or absence of *Sli* either, as postulated by Sanetomo et al. [29]. As observed by Hosaka and Hanneman [36,37] *Sli* inhibits *S-RNase* function in the pollen tube, thus overcoming the prezygotic GSI barrier. Inability to produce viable seed after fertilization due to endosperm failure is a post-zygotic barrier, resulting from differences in effective ploidy [38,39]. These two interspecific barriers are independent even though the outcome is the same.

## 3. Discussion

The relationships of interspecific compatibility and incompatibility in *Solanum* section *Petota* are complex. Inquiry into these relationships has elucidated the pleiotropic and redundant function of *S-RNase* and *HT* which tandemly and independently mediate both interspecific and intraspecific pollen rejection [15,20,23]. The outcome of interspecific pollinations is specific to the individual species involved and direction of the cross due to the direct and indirect involvement of multiple factors such as *HT* and *S-RNase*, 120 kDa, Cullin1 (*CUL1*), and farnesyl pyrophosphate synthase (*FPS2*) [16,20,40,41].

The findings presented here are consistent with previous work conducted in *Solanum* section *Lycopersicon* showing that *S-RNase* plays a central role in interspecific pollen rejection [15,23]. Statistical analyses also demonstrated that *HT-B* alone is not a significant factor in these pollinations. These results do not contradict the previous findings from tomato, where HT proteins played a significant role in interspecific pollen rejection [20,23]. Since *HT-A* remained functional in all of the female genotypes used, these findings demonstrate the overlap in gene function between *HT-A* and *HT-B*, the greater importance of *HT-A*, or that *HT* is not a significant factor in these combinations, implicating other mechanisms [15,33].

In comparison to the other species used as pollen donors, *S. commersonii* was unique as the absence of *S-RNase* did not appear to affect the rejection of pollen in *S. tuberosum*. Since the rejection or acceptance of interspecific pollen is entirely dependent on the specific combination of species used as parents, *HT*, *S-RNase*, and other factors may play significant or inconsequential roles during the rejection of interspecific pollen [15,16]. In this instance *HT-A* alone may mediate the *S-RNase* independent rejection of *S. commersonii* pollen in DRH195.124-001, where both *HT-B* and *S-RNase* are non-functional. If this is the case, this would be consistent with the findings of Tovar-Méndez et al. [20].

Prezygotic stylar barriers were not observable in these pollinations using *S. verrucosum* as the female parent. The lack of prezygotic stylar barriers in *S. verrucosum* may be comparable to *S. lycopersicum* [15]. This lack of IRBs is a very valuable phenotype which allows *S. lycopersicum* to not only be SC, thus allowing the development of inbreds and F1 hybrids, but also serves as a parent for wide interspecific crosses allowing the introgression of valuable traits from genetically distant wild relatives [15,42,43]. The lack of SC and interspecific incompatibility in diploid *S. tuberosum* has been a significant barrier to the effective use of wild species, and the ability to replicate the phenotype observed in *S. verrucosum* and *S. lycopersicum* within *S. tuberosum* would be a significant advancement in potato breeding [7,12]. Understanding the causative genetic mechanisms behind SC and interspecific compatibility in *S. verrucosum* is necessary to replicate this phenotype. Additionally, stylar barriers are not the only significant factors barring the use of wild potato species in potato breeding. Therefore, further inquiry into the genetic mechanisms underlying effective ploidy and endosperm balance number will be necessary in the future.

Although *Sli* played no significant role in these pollinations, it is possible that *S. chacoense* as a pollen donor is better able to bypass stylar barriers in EBN1 species such as *S. pinnatisectum* compared to *S. megistacrolobum*, *S. demissum*, and *S. phureja* (*S. tuberosum* group *Phureja*), as reported by Sanetomo et al. [29]. Consequently, *S. chacoense* may be a valuable resource in accessing these EBN1 species regardless of *Sli* status. Currently, other methods such as bridge crossing with *S. verrucosum* are more reliable and feasible in their ability to access 1EBN species [2]. Unlike the methods proposed by Sanetomo et al. [29] and Carputo et al. [44], the use of *S. verrucosum* bridge crosses is also more accessible for most plant breeders, as it does not require ploidy manipulation, interploidy crosses, or the use of advanced techniques [2].

## 4. Materials and Methods

### 4.1. Plant Material

The wild species *S. bulbocastanum*, *S. commersonii*, *S. jamesii*, and *S. pinnatisectum* were used as both pollen donors and females for this study. Multiple plant introduction populations for each species were acquired from the USDA-ARS Potato Germplasm Introduction Station (Sturgeon Bay, WI, USA), and individual genotypes were selected from their respective field-grown plant introduction populations based on their fertility and overall vigor (Table 1). In this study, *S-RNase* and/or *HT-B* mediated interspecific pollen rejection was investigated using CRISPR-Cas9 gene knockouts of *S. tuberosum* clone DRH195 and untransformed controls (Table 1) [12,26]. Interspecific compatibility in *S. verrucosum* was investigated using the clone SV607845.02. This *S. verrucosum* clone was selected as a female based on its previous ability to create interspecific hybrid offspring with *S. bulbocastanum*. The *S. chacoense* clone USDA8380-1 and the *S. chacoense* self-compatible inbred line M6 were selected as pollen donors to evaluate *Sli* [34]. The M6 clone was selected as it is a homozygous, well characterized, and widely used source of *Sli*-based SC [32,34]. In order to reduce confounding genetic variables, clone USDA8380-1 was selected as the non-*Sli* control as it is largely homozygous, and also the same species as M6 [35]. Each genotype was maintained in tissue culture on Murashige and Skoog Basal Medium with Vitamins and Sucrose (PhytoTech Labs, Lenexa, KS, USA) and Phyto Agar (Research Products International, Mount Prospect, IL, USA) prepared with DI water and balanced with 1M HCl and 8N NaOH to a pH of 5.8 and cultured in growth chambers with 16-h-light/8-h-dark photoperiod at 22 °C and average light intensity of 200 μmoles m^−2^s^−1^.

### 4.2. Greenhouse Pollination Assays

In October of 2020, 2 individuals from each genotype were transferred directly from tissue culture to 14 L (3.8 gallon) plastic pots filled with Suremix Perlite peat and perlite soilless medium (Michigan Grower Products Inc., Galesburg, MI, USA). For the duration of the experiments greenhouse conditions were maintained at 20 °C with a 16-h photoperiod under Philips GreenPower light-emitting diode (LED) DR/W-MB lights (Philips Lighting Holding B.V., Eindhoven, The Netherlands). To validate the male fertility of the pollen donors, pollen was collected from 5–6 anthers directly onto a glass slide and immediately stained with acetocarmine-glycerol as described by Ordoñez [45], covered with a cover slip, and sealed using clear nail polish. These slides were stored at room temperature in the dark and visualized the same day using a Leica DM750 binocular microscope (Leica Microsystems, Wetzlar, Germany) and the associated Leica imaging software at 10× and 40× magnification. A minimum of 100 pollen grains were then used to calculate the percentage of viable pollen. Stained, turgid pollen was classified as viable, while any grains that were shriveled, unstained, or unusually large or small were classified as unviable.

### 4.3. Stylar Squash Assays

For the evaluation of stylar barriers in interspecific crosses, 6–8 newly opened flowers (within 24 h after anthesis) from each of the female genotypes were carefully emasculated and pollinated with fresh pollen collected on a glass slide directly from the pollen donor. Styles were collected 48 h post-pollination, by removing the remaining petals and sepals and storing the remaining intact style, ovary, and receptacle in a 1.5 mL microcentrifuge tube containing a 3:1 ethanol/acetic acid fixation solution. These styles were then kept in the dark at room temperature for at least 24 h. Styles and ovaries were then softened using an 8N NaOH solution at 60 °C for 1 h. Samples were then rinsed 3 times with distilled water and stained with 0.1% aniline blue in 0.1N K_3_PO_4_ keeping them in dark conditions with light shaking. Styles with attached ovaries were then placed on glass slides, gently squashed under a coverslip, and sealed with nail polish for subsequent visualization. Samples were visualized using a Nikon Eclipse Ni-U upright microscope (Nikon Instruments Inc., Melville, NY, USA) with a SOLA light engine (Lumencor, Beaverton, OR, USA), and photographed with the attached ANDOR Zyla sCMOS camera (Oxford Instruments, Abingdon, UK) and NIS-Elements BR 5.02 software. Images of each stylar sample were stitched together using Image Composite Editor 2.0 software. Pollen tube growth measurements were made using ImageJ 1.53e software [46]. Measurements of pollen tube growth were calculated as a proportion of the total length of the style due to the variation of total style length with a single genotype. Pollinations were made at 3 discrete time points, separated by 30 days, with the first time point being approximately 6 weeks after plants were transferred from tissue culture to the greenhouse.

### 4.4. Data Collection and Analysis

**Figure 5:**
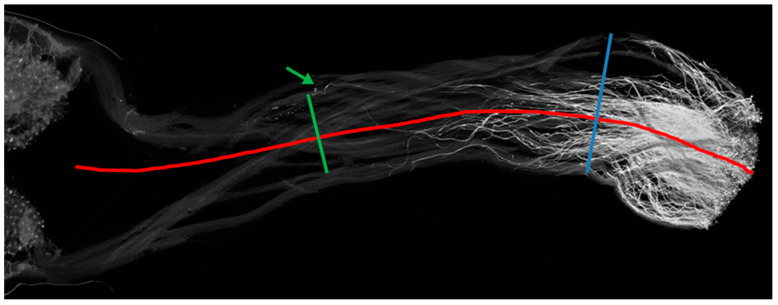
Pollen tube measurement description (style of WT DRH195 with *S. pinnatisectum* as pollen donor). Total length of the style was measured along the midline of the style from the stigma surface to the base of the style, following the curvature if present (depicted by the red line). The pollen tube front was measured along the midline, at the point where the majority of the pollen tubes stopped (Blue line). The measurement would be taken at the point where the blue and red lines intersect. The longest pollen tube (identified by the green arrow) was measured along the midline and would be taken where the green and red line intersect.

Measurements of the pollen tube front, or the point where the majority of the pollen tubes stop, the longest pollen tube, and the total length of the style were collected using ImageJ software (Figure 5). From these measurements, distance from the surface of the stigma to the pollen tube front and length of the longest pollen tube were calculated based on the total length of the style in which they were measured (Supplemental File S1). Significant differences between means (α = 0.05) from three replicated measurements were calculated using analysis of variance (ANOVA) and Tukey’s honestly significant difference (HSD) using “R” software version 4.0.4.

## Figures and Tables

**Table 1:** List of plant material used as pollen donors and females.

Line ID	Species	PI *	Gene Edit
SBGG505-A	*S. bulbocastanum*	275197	-
M6	*S. chacoense*	BS 228	-
USDA8380-1	*S. chacoense*	458310	-
Scmm320266-02	*S. commersonii*	320266	-
SPGG544-A	*S. jamesii*	592417	-
SJGG520-A	*S. pinnatisectum*	275232	-
SV607845.02	*S. verrucosum*	607845	-
DRH195	*S. tuberosum*	-	-
DRH195.158	*S. tuberosum*	-	*S-RNase* KO
DRH195.121_009	*S. tuberosum*	-	*HT-B* KO
DRH195.124_001	*S. tuberosum*	-	*S-RNase*/*HT-B* KO

* Plant introduction number issued by the U.S. National Plant Germplasm System (NPGS).

## Data Availability

Data are contained within the article and Supplementary Materials.

## Supplementary Materials

The following supporting information can be downloaded at: https://www.mdpi.com/article/10.3390/plants12081709/s1, Supplementary File S1: Pollen tube growth measurements.
